# To: Diagnostic accuracy of lung ultrasound in children with respiratory pathology admitted in a pediatric intensive care unit of a low-resource setting: a single-center experience

**DOI:** 10.62675/2965-2774.20260154

**Published:** 2026-08-31

**Authors:** Mohd Saif Khan, Anis Chaari

**Affiliations:** 1 King Hamad University Hospital Intensive Care Muharraq Bahrain Intensive Care, King Hamad University Hospital - Muharraq, Bahrain.

**Dear Editor,**

We read with interest the article "Diagnostic accuracy of lung ultrasound in children with respiratory pathology admitted in a pediatric intensive care unit of a low-resource setting: a single-center experience".^(1)^ The study robustly addresses a pertinent issue in resource-limited pediatric intensive care units (ICUs), but several methodological and interpretive issues warrant consideration.

**Major concerns:** a central limitation is the choice of chest X-ray as the gold standard for diagnostic accuracy. Chest X-ray is itself an imperfect comparator, with limited sensitivity for small effusions, pneumothorax, and early pneumonia. Reliance on chest X-ray likely contributed to the poor agreement observed (kappa = 0.085) and undermines the strength of conclusions regarding lung ultrasound accuracy. A composite clinical diagnosis or computed tomography imaging, though less feasible, would have provided a more reliable reference.

The study reports sensitivity and specificity values but does not sufficiently discuss their clinical implications. For example, what does a specificity of 19% mean for bedside decision-making in a low-resource pediatric ICU? This gap leaves readers uncertain about how to apply the findings in practice. Moreover, such a poor specificity undermines the claim that lung ultrasound could be a reliable diagnostic tool risking over-diagnosis and unnecessary interventions.

Only one sonographer performed all scans after a brief 1-day training plus 3 supervised days. This raises concerns about operator dependency and reproducibility.

The study design also introduces potential bias. Only patients who underwent chest X-ray were included, which may have excluded children with milder disease or those managed without radiography. The exclusion of patients without chest X-ray introduces selection bias. Children stable enough for both imaging may not represent the sickest cohort. High proportion of infants (< 12 months) complicates generalizability, as acoustic windows and pathology prevalence differ by age.

**Minor concerns:** although Bayesian methods were applied, their results are not well integrated into the discussion. The added value of Bayesian analysis – particularly in small or imbalanced samples – should be explained more clearly. Otherwise, readers may see it as an unnecessary methodological add-on. The research could better position itself against prior pediatric ICU studies.^(2,3)^ The discussion cites Tripathi et al. and DeSanti et al. studies but does not adequately explore why their results differ (e.g., operator experience, case mix, and definitions). This weakens contextualization. A clearer statement of how this study adds to existing literature would strengthen its impact.

**Interpretation:** the authors conclude that lung ultrasound is feasible but of limited diagnostic accuracy compared to chest X-ray. While feasibility is demonstrated, diagnostic performance is highly variable across conditions. Sensitivity and specificity were moderate for pediatric acute respiratory distress syndrome (72.7% and 95.7% *versus* clinical diagnosis), and suboptimal for pneumonia (59.8% and 54.3%). These findings suggest that lung ultrasound may serve as an adjunct rather than a replacement for chest X-ray in a low-resource pediatric ICUs.

**Conclusion:** this study adds important evidence from a low-resource setting, but reliance on chest X-ray as the gold standard, low specificity, and selection bias weaken the strength of its conclusions. Future multicenter studies with broader inclusion criteria, standardized training, and composite diagnostic references are needed to clarify the role of lung ultrasound in pediatric critical care.
